# Stretching Interventions in Children With Cerebral Palsy: Why Are They Ineffective in Improving Muscle Function and How Can We Better Their Outcome?

**DOI:** 10.3389/fphys.2020.00131

**Published:** 2020-02-21

**Authors:** Barbara M. Kalkman, Lynn Bar-On, Thomas D. O’Brien, Constantinos N. Maganaris

**Affiliations:** ^1^School of Sport and Exercise Sciences, Liverpool John Moores University, Liverpool, United Kingdom; ^2^Department of Rehabilitation Medicine, VC University Medical Center Amsterdam, Amsterdam, Netherlands

**Keywords:** muscle, tendon, *in vivo*, sarcomere addition, stiffness

## Abstract

Hyper-resistance at the joint is one of the most common symptoms in children with cerebral palsy (CP). Alterations to the structure and mechanical properties of the musculoskeletal system, such as a decreased muscle length and an increased joint stiffness are typically managed conservatively, by means of physiotherapy involving stretching exercises. However, the effectiveness of stretching-based interventions for improving function is poor. This may be due to the behavior of a spastic muscle during stretch, which is poorly understood. The main aim of this paper is to provide a mechanistic explanation as to why the effectiveness of stretching is limited in children with CP and consider clinically relevant means by which this shortcoming can be tackled. To do this, we review the current literature regarding muscle and tendon plasticity in response to stretching in children with CP. First, we discuss how muscle and tendon interact based on their morphology and mechanical properties to provide a certain range of motion at the joint. We then consider the effect of traditional stretching exercises on these muscle and tendon properties. Finally, we examine possible strategies to increase the effectiveness of stretching therapies and we highlight areas of further research that have the potential to improve the outcome of non-invasive interventions in children with cerebral palsy.

## Introduction

Cerebral palsy (CP) is a non-progressive disorder caused by a brain lesion taking place in the early stages of development (Graham et al., 2016). The neurological lesion in CP causes adaptations in the muscle, including muscle atrophy (Shortland, 2009), fibrosis (Booth et al., 2001), muscle shortening (Barrett and Lichtwark, 2010) and overstretched sarcomeres (Mathewson et al., 2014). The combination of longer sarcomeres with shorter muscle fibers mean that there will be fewer in-series sarcomeres (Mathewson et al., 2014). Additionally, there is a lack of muscle growth (Gough and Shortland, 2012; Willerslev-Olsen et al., 2018). This dynamic shortening of the muscles is typically treated with stretching exercises, botulinum toxin injections, casting or ankle-foot-orthoses. Eventually, if these treatments are not sufficiently effective fixed contractures can develop, which is treated with surgery.

One of the main aims of these treatments which try to maintain or increase ROM is to improve the gait pattern. An increase in muscle-tendon unit (MTU) length could be gained by permanently stretching existing structures, however, this is probably less desired as a carry-over effect to function may not occur. To improve functionality, the increase in length should be rather achieved by building new contractile material in-series within the MTU. Ideally, we want to promote large, strong and flexible muscles that allow contractile function across the muscle’s full range of motion. Therefore, interventions should aim to increase fibers length by promoting serial sarcomerogenesis. According to the force-length characteristics of muscle (Lieber, 2002), such an increase in muscle fibers length would allow for force production over the newly acquired ROM.

For the purpose of increasing ROM, different stretching methods have been used. Passive or active stretches can be applied either manually by a clinician or by the patients themselves as initial conservative treatments. Ankle-foot-orthoses or casting are used to hold the joint near their end-range of motion to provide a more chronic stretch of the muscle. By increasing ROM, stretching methods should consequently better daily function, delay the development of contractures and the need for surgical intervention This relies on the assumption that the muscle is able to generate forces throughout a larger excursion used over the increased ROM. The stretching exercises can cause discomfort to the children, they are time consuming for the patient their families (Hadden and Von Baeyer, 2002) and the physiotherapists (Wiart et al., 2008). Additionally, there is limited evidence to support functional improvements, e.g., in gait and mobility, after stretching interventions (Preissner, 2002; Katalinic et al., 2011; Harvey et al., 2017). Clearly a significant gap exists between the clinical rationale for implementing stretching and the supporting evidence for its intended effectiveness. Despite this, stretching remains a widely prescribed method of treatment, probably due to the comparative simplicity and safety of its application as a first line treatment. Therefore, the main aim of this paper is to review the evidence and provide a mechanistic explanation as to why the effectiveness of stretching is limited in children with CP and consider clinically relevant means by which this shortcoming can be tackled.

To do this, we will first discuss how the muscle and its tendon interact in children with CP based on their morphology and mechanical properties to limit ROM at the joint. This discussion will be presented in terms of a simplified muscle model of the medial gastrocnemius. The discussion will focus on the contribution of different structures within the MTU to the ROM and consider whether the fibers experience an adequate stretching stimulus to promote adaptation. Second, we will consider the effect of traditional stretching exercises on the muscle and tendon properties in children with CP by examining the literature about both manual passive stretching, ankle-foot-orthoses and lower-leg casts on muscle and tendon properties. Finally, we will examine possible solutions to increase the effectiveness of stretching therapies and highlight areas of further research that have the potential to improve the outcome of non-invasive interventions for children with CP.

## Muscle and Tendon Properties in CP

**FIGURE 1:**
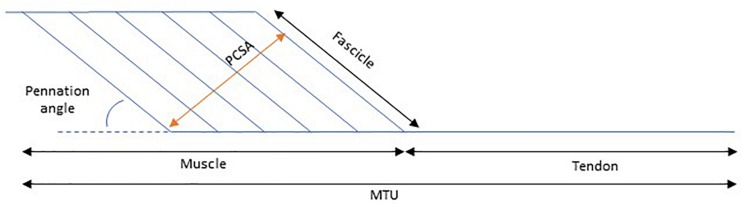
Schematic representation of the contribution of muscle length to muscle tendon unit (MTU) length. PCSA, Physiological cross-sectional area.

The mechanical properties of a muscle dictate the degree of its length alteration in response to an applied force. This response depends partly on the geometry of the muscle, as well as on the intrinsic tissue material properties. In this paper we will discuss the changes that occur to the architecture and the mechanical properties of the medial gastrocnemius muscle in children with CP. To do this, it is common to use a simplified pennate muscle model (Figure 1) of the medial gastrocnemius. In this model, the MTU consists of a muscle and a tendon placed in series, where the changes in the muscle belly length are dependent on changes of the muscle fascicles and the pennation angle. The medial gastrocnemius muscle is most commonly studied in children with CP for a few reasons: it is a superficial muscle, therefore suitable for most imaging techniques and this muscle is essential for functional tasks such as walking. The architectural structure of muscles can be studied in a straightforward manner with medical imaging techniques such as MRI (e.g., Elder and Kirk, 2003), and 2D (e.g., Shortland et al., 2002) or 3D ultrasound (e.g., Cenni et al., 2018a; Cenni et al., 2018b, while the mechanical properties (stiffness) of a muscle can be assessed by applying a known tensile force to the tissue and measuring the resultant elongation (Maganaris, 2002). Due to anatomical constraints, some assumptions need to be made when quantifying *in vivo* muscle and tendon stiffness. For example, when applying a passive moment around the ankle joint to dorsiflex the foot, the lengthening of the medial gastrocnemius MTU achieved depends on the MTU’s moment arm length (Kalkman et al., 2017) the properties of all agonist plantarflexor and antagonist dorsiflexor MTUs acting around the ankle joint, and the properties of all passive structures within the joint (e.g., ligaments and joint capsule) These factors might vary with ankle angle, making it difficult to quantify the passive force carried by MTU during passive joint rotation. Alternatively, instead of calculating muscle stiffness in absolute terms, we can estimate the relative contribution of muscle and tendon to the joint’s ROM as the muscle and its tendon are placed in series and therefore the passive force along these two structures is expected to be the same.

### Muscle Architecture in CP

There is consistent evidence that the medial gastrocnemius muscle is shorter in the paretic leg of children with CP compared to matched typically developing children (Fry et al., 2007; Malaiya et al., 2007; Barrett and Lichtwark, 2010). This is accompanied by a greater length of the Achilles tendon in children with CP (Wren et al., 2010; Barber et al., 2012). Furthermore, some studies have reported reduced muscle fascicle lengths at rest in children with CP than in TD children (Mohagheghi et al., 2008; Matthiasdottir et al., 2014; Kalkman et al., 2018b; D’Souza et al., 2019; Frisk et al., 2019), but others have not detected differences (Shortland et al., 2002; Malaiya et al., 2007).

These findings can have significant functional consequences. Shorter muscle fascicles with fewer sarcomeres in series, as found in children with CP (Lieber and Fridén, 2002; Ponten et al., 2007; Mathewson et al., 2014), will have a limited potential for active shortening. This, would in turn influence the force generating capacity of the muscle (Frisk et al., 2019). It has also been shown that the force-length relation of muscles in children with CP was different to that of typically developing children, with maximal torque generation occurring at a more plantar flexed position (Frisk et al., 2019). This may be a contributing factor to gait impairments such as toe-walking, the more plantarflexed joint position during gait would take advantage of the altered moment-angle relationship. These findings highlight that it is essential to increase fibers length and extensibility to improve the force-length relation of the muscle, firstly with respect to the position at which maximal torque is produced and secondly with respect to the range over which force can be produced (Lieber and Friden, 2000).

### Lengthening Behavior of the Muscle

For an increase in fibers length to occur in response to a stretching intervention, it is essential that the muscle fibers receive an adequate stretching stimulus. The amount of stretch that the muscle experiences is dependent on the mechanical properties of both the contractile tissue as well as the connective tissue within the muscle fibers and the tendon. It has been shown that, when the ankle joint is passively dorsiflexed *n* children with CP, the belly of medial gastrocnemius muscle elongates less compared to TD children (Matthiasdottir et al., 2014). Additionally, some studies have reported lengthening of the muscle fascicles to be unaltered during passive joint rotation (Matthiasdottir et al., 2014), while others show a reduction in fascicle lengthening in CP when compared to TD children (Barber et al., 2011; Kalkman et al., 2018b). These inconsistent findings could potentially be accounted for by different ways of comparing groups. A reduced ROM in children with CP could complicate comparisons made over the full ROM. Additionally, due to differences in Achilles tendon moment arm length (Kalkman et al., 2017) and joint stiffness (Alhusaini et al., 2010) between TD and CP participants, comparisons in terms of joint angles should be interpreted with caution. To circumvent this problem, fascicle lengthening during passive joint rotations has been additionally studied over a common torque range and a common MTU lengthening, where it was found that irrespective of the method used the stiffness of the muscle fascicles relative to their in-series tendon was higher in children with CP (Kalkman et al., 2018b).

Due to the above-mentioned changes in muscle and tendon properties, the stretch “seen” by the muscle and the fascicles would be reduced in children with CP. It has been shown in rabbits that strain experienced by the muscle fibers is a more potent stimulus for in series sarcomerogenesis than MTU strain (Butterfield and Herzog, 2006). Therefore, it is reasonable to assume that the reported alterations to muscle and tendon properties play a crucial role for the response to stretching interventions, as is expected in TD individuals (Morse et al., 2008).

## The Effect of Stretching on Muscle and Tendon Properties in Children With CP

Muscle stretching, in the form of passive stretching exercises, orthotics, casting, standing tables, or a combination of these modalities, has been recommended in the early management of joint hyper-resistance in children with CP (Pin et al., 2006; Blackmore et al., 2007; Bovend’Eerdt et al., 2008; Novak et al., 2013). Here we assess the effect of traditional stretching exercises on muscle and tendon properties. Although there is an abundance of literature showing a positive effect of stretching exercises on ROM [as reviewed by Eldridge and Lavin (2016)], functional indexes such as gait parameters, walking velocity and gross motor function rarely improve (Craig et al., 2016). This may be because the muscle and the tendon do not adapt in the required ways, a concept that has not been studied systematically.

During typical growth and development there is an age-dependent adaptation of muscle and tendon in response to stretch. It has been suggested that during normal growth, both the muscle and the tendon grow, and the tendon grows at a slightly lower rate (Wren, 2003). Younger animals have been shown to adapt to immobilization by increasing tendon length, where older animals add sarcomeres in-series (Tardieu et al., 1977). The use of a computational model suggested that the tendon acts like this at young age in order to minimize tensile strains (Wren, 2003). Growth factors that are altered during development may be responsible for these different muscle and tendon adaptation across the lifespan (Okamoto et al., 2005). In children with CP this is additionally complicated by the fact that growth factor seems to be further altered from typical (Von Walden et al., 2018; Pingel et al., 2019). This raises the question whether it is the muscle or the tendon adapting in younger and older children with CP. Are the muscles able to increase length by sarcomerogenesis or is the tendon additionally lengthened by stretching treatments?

### Passive Stretching

Passive stretching exercises are carried out by a therapist without the patient’s own muscular involvement. The muscle to be stretched is lengthened as the therapist manually moves the joint of the patient at its end-range position. The therapist holds this end-range position for a certain amount of time. The effect of manual passive stretching on muscle and tendon properties has been investigated both acutely after a bout of stretching and after a long-term intervention. Acutely, passive stretching has been shown to increase ROM in children with CP (Theis et al., 2013; Kalkman et al., 2018a). Theis et al. (2013) performed passive stretches for five repetitions of 20 sec, either applied by a physiotherapist or by the children themselves. Kalkman et al. (2018a) performed three sets of five 20 sec repetitions of passive stretching, all applied by a physiotherapist. According to Theis et al. (2013) the increase in ROM was accounted for by an increase in length of all 3 structures that make up the MTU, muscle belly, fascicles and tendon. However, the average reported increase in MTU lengthening after stretching (18.5 mm) seemed extremely large for an increase in ankle ROM of only 9.8°. A later study showed that a similar increase in ROM (10°) achieved directly after a single bout of stretching resulted in only 3.9 mm average increase in MTU lengthening, where 80% of the increase in maximal MTU length was accounted for by the muscle fascicles and the remaining 20% could be attributed to the Achilles tendon and aponeurosis (Kalkman et al., 2018a). Kalkman et al. (2018a) additionally showed that the amount of length change in the muscle belly achieved by joint rotation over a common ROM was not altered. Therefore, they concluded that the increased ROM could be due to a greater torque applied passively by the examiner and thus an increased tolerance to stretch, dependent on several factors, including, pain tolerance, warm-up, and acquaintance between therapist and patient.

After a 6-week stretching intervention that consisted of 15 min stretching (10 repetitions of 60 sec) for four times a week, it was shown that ROM increased, joint stiffness decreased, resting fascicle length remained similar and muscle stiffness decreased (Theis et al., 2015). However, as mentioned above there are several limitations associated with measuring muscle stiffness, since the calculated force in the muscle depends on the MTUs moment arm, passive structures around the joint and other agonist/antagonist muscles. Nonetheless, the authors hypothesized that the changes in stiffness were due to changes in muscular connective tissue and not by an increase in serial sarcomere number (Theis et al., 2015). A similar 6-week stretching intervention was performed by Kalkman et al. (2019), who found an increase in ROM and no changes in resting fascicle length. Hence, similarly to the acute response (Kalkman et al., 2018a), it was hypothesized that the increase in ROM was caused by a chronic increase in stretch tolerance. This concept would also be able to explain the increase in fascicle strain as seen in Theis et al. (2015). Contrasting results were found by Hösl et al. (2018) who reported a small decrease in resting fascicle length after 9 weeks of passive stretching. However, fascicle strain did increase, which was attributed to larger passive dorsiflexion ROM and tolerated stretch-moments (Hösl et al., 2018). In comparison to Theis et al. (2013) and Kalkman et al. (2018a) the time period each stretch was held was shorter (20 s vs. 60 s), but the total intervention time was about 27% longer. The literature is still lacking studies looking at changes in the structure of individual muscle fibers (at sarcomere level) after stretching interventions, which could possibly clarify and explain the observations described above.

### Ankle Foot Orthoses

An ankle foot orthosis (AFO) is a medical device that imposes a mechanical constraint to the ankle and the foot. The AFO can directly affect movement of the ankle both during gait and at rest (i.e., at night). AFO’s are commonly used to hold the joint more permanently near the end range of motion applying constant, moderate stretch to the MTU. The only study found that reported the effect of AFO’s on muscle/tendon structure in children with CP, showed little change in muscle belly and tendon length after 16 weeks of ankle-foot bracing (Hösl et al., 2015). However, a 11% decrease was found in fascicle length, which was suggested to be caused by a loss of sarcomeres in series (Hösl et al., 2015). Similar to the results described in the previous section, it seems that the muscle does not “see” much of the stretch stimulus in AFO’s too, which might explain these findings of limited changes in muscle/tendon structure. The authors concluded that it seems difficult to change muscle morphometrics by single traditional treatments and there is a need for concomitant treatments to promote muscle growth (Hösl et al., 2015). Additionally, a simulation study showed that the operating length of the medial gastrocnemius MTU during gait is highly variable between individuals and types of AFO (Choi et al., 2016), suggesting that, with a proper orthotic design, it may be possible to promote in series sarcomerogenesis. Clearly, more focused research is required to identify the mechanisms of inter-subject variability in muscle remodeling in response to chronic stretching.

### Serial Casting

Lower-leg casting refers to the application of plaster to immobilize the ankle joint. Serial or progressive lower-leg casting involves the successive application of a series of casts, placing the foot at gradually increasing dorsiflexion angles with each cast. In an initial study, which still requires full review, publication, and confirmation, the effects of 2 weeks of lower-legs casting only on the ankle joint as well as the underlying muscle and tendon properties, was examined in a group of children with CP (Peeters et al., 2018). It was shown that post-casting, an increased maximum ankle dorsiflexion angle was accompanied by increased tendon length, rather than muscle length (Peeters et al., 2018). More studies are needed, especially to evaluate if the effects may differ depending on age, as shown in animals (Tardieu et al., 1977). If corroborated, these results will also be in line with the findings described above where the increase in joint ROM after the use of AFO’s could be explained by an increase in tendon length (Hösl et al., 2015). Once again, this effect may be explained by the same principle applied during passive stretches, where the tendon “sees” most of the stretch (Kalkman et al., 2018b).

## Improving the Effectiveness of Stretching in CP

As discussed above, stand-alone stretching methods applied manually, with AFO’s or serial casting do not seem effectively improve the muscle and tendon. Adaptations seem to be either absent or in a negative direction after a period of stretching. In this context, a combination of treatments have been tried to increase the stretch stimulus to the muscle. In this section we will discuss the effect of different combination treatments on muscle and tendon properties.

### Botulinum Toxin-A and Stretching

Common in clinical practice is the combination of Intramuscular Botulinum neurotoxin-A (BoNT-A) injections with stretching by serial casting. The hypothesis is that BoNT-A injections temporarily reduce the symptoms of excessive tonic discharge thus providing a “window of opportunity” during which adjunctive interventions such as serial casting and physiotherapy can be implemented to improve, or at least prevent further deterioration of, muscle structure and functionality (Franki et al., 2019). Therefore, when these are reduced, the muscle will experience more of the tensile stimulus applied by the adjunctive stretching intervention. The above reasoning goes hand in hand with the idea that contracture is due primarily to hyperactive stretch reflexes. However, the evidence for that is quite the contrary, with muscle alterations preceding the emergence of hyperactivity (Willerslev-Olsen et al., 2013, 2014). Nevertheless, it is assumed that when hyperactive stretch reflexes are reduced, the muscle will experience more of the tensile stimulus applied by an adjunctive stretching intervention. There is limited evidence that BoNT-A combined with rehabilitation is effective in altering muscle structure or preventing contracture (Boyaci et al., 2014), even though there are indications of short term improvement in joint ROM, gait and function, as measured by the goal attainment scale (Molenaers et al., 2013; Novak et al., 2013). Specifically, promising results have been observed in functional improvements, as measured by the Goal Attainment Scale, after combining BoNT-A with different kinds of stretching therapies (Molenaers et al., 2013). More research is needed to better understand how these functional improvement are achieved.

Evidence from animal studies suggest that BoNT-A may increase muscle stiffness, and that its long term use may cause detrimental levels of muscle atrophy (Gough et al., 2007; Pingel et al., 2016). Additionally, it was shown that muscle volume decreased significantly after BoNT-A injections (Williams et al., 2013b), usually an unwanted outcome that could be counterbalanced with strength training (Williams et al., 2013a). Together with a reduction in normalized muscle volumes, alterations to muscle quality were shown by a reduction in echogenicity in a cohort of children with who had received BoNT-A injections compared to a cohort of children with CP who did not receive any BoNT-A injections (Schless et al., 2019). Although no difference could be shown between single and multiple BoNT-A injections (Barber et al., 2013), it seems unlikely that multiple BoNT-A injections would promote muscle growth, the desired outcome from the stretching interventions discussed in this review.

### Specific Collagenase Enzymes and Stretching

To avoid the negative effects of BoNT-A injections on the muscle, another way to reduce the stiffness of the spastic muscle has recently been proposed. It was hypothesized that injecting specific collagenase enzymes, to digest part of the extensive collagen in the extracellular matrix (Smith et al., 2011), which play a role in contractures would reduce muscle stiffness (Howard et al., 2019). This theory is about to be tested in a spastic mouse model, but it is hypothesized that if selective collagenase is injected into spastic muscle at an appropriate dilution and concentration in combination with a stretching program, it might lead to increased ROM and improvements in sarcomere length, hence enhancing force production over the newly acquired ROM (Howard et al., 2019).

### Strengthening and Stretching

**FIGURE 2:**
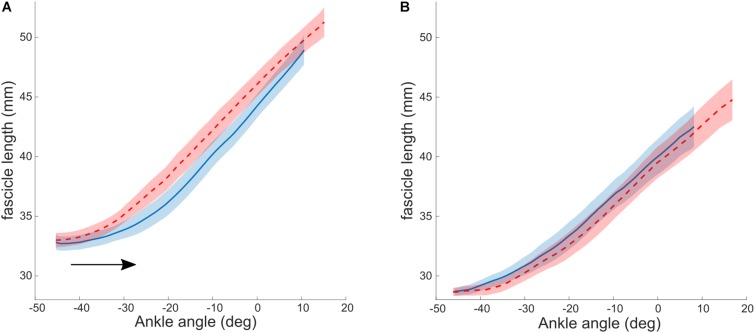
Lengthening profiles of muscle fascicles vs. ankle angle during a passive joint rotation in the intervention **(A)** and control **(B)** group. Negative angles reflect plantarflexion position. The black arrow indicates the shift in ankle angle at which the fascicles start to lengthen. Blue: baseline, red: after 10 weeks of intervention. Values are reported as the median and interquartile range (IQR). Reused from Hösl et al. (2016), held under CC-BY 4.0.

As described above, the aim of a combined treatment is to increase the stretching stimulus to the muscle. BoNT-A injections achieve this by a reduction in tonic discharge, that alters the relative lengthening of muscle and tendon during stretch (Bar-On et al., 2014). Alternative methods for the same purpose, have been proposed. For example, the relative lengthening of muscle to tendon during passive stretch could be increased when tendon stiffness is increased. It has been shown that tendon stiffness is adaptable to mechanical loading and can increase with resistance training in several populations, including adults (Couppé et al., 2008), elderly (Reeves et al., 2003) and typically developing pre-pubertal children (Waugh et al., 2014). A combined strengthening and stretching intervention was designed by Kalkman et al. (2019) to promote the stretch seen by the muscle. This intervention group was compared to a control group, who performed only stretching exercises. Heel raises were performed as progressive resistance training exercises to strengthen the ankle plantar flexor muscles. The target was to complete 3 sets of 12 repetitions four times a week, exercises were progressed by adding weight to a backpack. Muscle strength, as measured by a maximal voluntary contraction (MVC), increased significantly, as did Achilles tendon stiffness. As a result of this, the stretching stimulus to the muscle was increased. Consequently, resting fascicle length in the intervention group increased (average of 2.2 mm), while in the control (stretching only) group it remained unaltered (average change of −0.5 mm). The authors thereby provided proof of concept that combining resistance with stretching training might be an effective intervention for increasing muscle fascicle length in children with CP. Additional analysis of fascicle lengthening during passive ankle rotation showed no pre to post changes in the control group. In the intervention group, however, there was a post-intervention shift toward greater dorsiflexion in the ankle angle at which the fascicles started to elongate when the ankle was moved passively (Figure 2). This is consistent with the fascicle length adaptation achieved and reinforces the notion that by increasing the stiffness of the tendon, the in-series fascicles can “see” more of the tensile stimulus applied during passive stretching and therefore adapt accordingly. However, the ROM improved similarly in the control and intervention groups, suggesting that stretch tolerance plays a more important role in limiting end range joint positions compared to in-series tissue mechanical properties. Functional benefits of an increased resting fascicle length on force production over the newly acquired ROM should be systematically and more directly investigated in the future.

Similar results were reported by Zhao et al. (2011) after passive-stretching and active-movement training. The training consisted of 20 min passive stretching, 30 min active movement and 10 min passive stretching. During the active movement training, a rehabilitation robot was used to actively engage the patients in computer games to exercise the ankle joint in both dorsi and plantar flexion directions. The passive stretching was performed by an ankle rehabilitation robot, which applied a predetermined torque to stretch the calf muscle toward end range dorsiflexion. After a 6-week training program, increases in Achilles tendon stiffness and medial gastrocnemius and soleus fascicle lengths across the ROM were reported. This study, however, does not report any comparisons to a control group, therefore it is difficult to draw conclusions about the mechanisms behind these observed changes.

### Electrical Stimulation and Stretching

A different approach to improve the efficacy of stretching interventions was suggested by Khalili and Hajihassanie (2008). It was proposed that electrical stimulation of the antagonist muscle may improve the efficacy of stretching by providing an additional tensile stimulus to the agonist muscle and by reciprocally inhibiting the stretched muscle. The intervention consisted of 30 min electrical stimulation of the quadriceps muscle three times a week and passive stretching of the hamstrings muscle five times a week. The intervention was applied to one leg, while the opposite leg served as a control receiving only passive stretching to the hamstrings. Maximal passive knee extension was shown to increase more in the intervention leg compared to the control leg. The mechanisms through which electrical stimulation might lead to the above improvements were not studied. Moreover, the technical complexity and discomfort associated with this treatment approach might prevent it from becoming routine clinical practice for ambulant children with CP.

### Eccentric Fascicle Loading

High mechanical stress and stretching to the muscle can also be achieved during eccentric strength training exercises. It has been shown that in healthy individuals eccentric plantarflexor training can promote strength and increase muscle fascicle length (Duclay et al., 2009). Backward-downhill treadmill training (BDTT) was suggested to provide such an eccentric fascicle loading on the gastrocnemius in children with CP (Hösl et al., 2016). Recently BDTT was compared to conventional stretching (Hösl et al., 2018). Passive calf stretches were performed for 5 × 20 s during 9 weeks by the control group. The intervention group walked backward downhill on the treadmill for 23 min, the speed and the slope of the treadmill were progressively increased and during the last 2 weeks participants had to carry weight belts to increase the load on the calf. No differences were found between the control and the intervention group in terms of muscle morphology or passive ROM. Small improvements were seen after BDTT in ankle dorsiflexion during comfortable walking speed and ambulatory mobility tests which could be a sign of altered neuromuscular control instead of changes in muscle structure.

### Difficulties of Muscle Remodeling

The question arising from all these studies is what the signaling pathways are behind the observed changes. Could spastic muscles be stimulated sufficiently to grow even though it has been suggested that growth factors are altered (Pingel et al., 2019), pro-inflammatory cytokines and reduced ribosomal production cause stunted growth (Von Walden et al., 2018) and a reduced skeletal muscle satellite cell number influences muscle morphology after chronic stretch in mice (Kinney et al., 2017). Some have raised the question whether remodeling of muscle might be difficult regardless of the treatment you perform. Nonetheless, promising changes have been observed due to some combination interventions described in this review.

## Conclusion

Although stretching may be beneficial to prevent worsening of muscle contractures (Wiart et al., 2008), as isolated treatments, they do not promote muscle length growth or improve function in children with CP. Alternative approaches to enhance the effectiveness of stretching exercises are needed. A few promising pathways have been highlighted in this review. Stretching combined with BoNT-A and electrical stimulation appear to have beneficial effects at the joint, but mechanistic work on whether this improved lengthening is provided from muscle or tendon is lacking and warranted. Active stretches (i.e., eccentric contractions) have been proposed as a “stretching alternative” and seem to promote gait and mobility parameters, but improvements in muscle properties are limited. Increasing tendon stiffness to enhance the stretching stimulus “seen” by the muscle seems to be a potentially promising strategy, as Kalkman et al. (2018a) found increases in fascicle lengths after this intervention and Zhao et al. (2011) reported similar results after an active movement and passive stretching intervention. However, more evidence is required to support this notion and additional studies are necessary to investigate the effectiveness of the combined treatments summarized above.

When studying the effect of (stretching) interventions in children with CP, the focus should be on the link between changes in ROM, the associated changes in muscle morphology, and whether these lead to improved muscle and mobility function. A central question that requires answering is: Would an increase in fascicle length lead to improved force production across the newly acquired ROM (Frisk et al., 2019) and does this lead to an improved gait pattern? Another point to consider is that none of the experimental studies discussed here have investigated the long-term effect of their proposed interventions. Since muscle contractures develop over time as children grow, an increase in fascicle length due to in-series sarcomerogenesis might not provide an immediate functional benefit, but it might prevent the development of fixed contractures at later age, thus delaying or possibly making corrective surgeries redundant. Another point to consider is the large inter individual variability in the cause of the increased resistance to stretch in muscles of children with CP. Recent research shows an interdependence between the amount of muscle lengthening and spastic reflexes during stretch (Bar-On et al., 2018). These results warrant individual assessment and treatment planning, taking into account the causes of the reduced ROM in children with CP.

## Author Contributions

BK, LB-O, TO’B, and CM have all contributed to different aspects in this manuscript.

## Conflict of Interest

The authors declare that the research was conducted in the absence of any commercial or financial relationships that could be construed as a potential conflict of interest.
